# A Service-Based Evaluation of a Therapist-Supported Online Cognitive Behavioral Therapy Program for Depression

**DOI:** 10.2196/jmir.2248

**Published:** 2013-06-27

**Authors:** John Sharry, Ruth Davidson, Orla McLoughlin, Gavin Doherty

**Affiliations:** ^1^Mater Misericordiae University HospitalDublinIreland; ^2^University of LimerickLimerickIreland; ^3^Counselling ServiceTrinity College DublinDublinIreland; ^4^School of Computer Science and StatisticsTrinity College DublinDublinIreland

**Keywords:** Internet, user-computer interface, depression, cognitive behavioral therapy, patient adherence, online interventions, guided online program, online therapist support, user experience

## Abstract

**Background:**

Evidence suggests that Internet-delivered cognitive behavioral therapy (CBT) may be as effective as face-to-face delivery for depression, but attrition and engagement rates remain a challenge.

**Objective:**

This service-based study aimed to evaluate an online, therapist-supported, CBT-based program for depression. The program was specifically designed to address engagement issues, most notably by integrating online therapist support and communication within the platform.

**Methods:**

Participants were 80 adults who were registered university students. Participants used the modular online program over 8 weeks, supported by a therapist. Engagement information was gathered automatically by the online system, and analyzed for all participants. Severity of participants’ self-reported symptoms of depression were assessed preintervention and postintervention using the Beck Depression Inventory-II (BDI-II). Postintervention measures were completed by 53 participants.

**Results:**

A high level of engagement was observed compared to a previous study within the same service, along with extensive use of a range of program features. A statistically significant (*P*<.001) decrease in self-reported depressive symptomatology from preintervention (mean BDI-II 25.47) to postintervention (mean BDI-II 15.53) with a large effect size (*d*=1.17) was also observed.

**Conclusions:**

The results indicate the potential of unintrusive and easily provided online support to enhance engagement with online interventions. The system described in the paper also illustrates how such online support can be tightly integrated with interactive online programs by using a range of design strategies intended to improve the user experience.

## Introduction

### Background

Many adults experience depression in their lifetime, but a relatively small number seek or receive effective or evidence-based treatment for their difficulties [1]. This may be due to a number of factors, including stigma associated with seeking help for mental health difficulties, a lack of knowledge about different treatment options, as well as logistical or fiscal reasons. Computerized or Internet-delivered treatment programs are the latest and most innovative approach to improving access to psychological treatments for mental health difficulties, including depression. A large number of programs have been developed and evaluated to date, targeting a variety of disorders, including depression, panic disorder, generalized anxiety disorder, phobias, obsessive compulsive disorder, eating disorders, and addictions [2]. Computerized programs have used a variety of therapeutic approaches, although cognitive behavioral therapy (CBT) is the most common treatment approach in such programs. CBT is perhaps particularly suited to adaptation to a computerized medium because of its well-structured protocol and clearly delineated activities and homework exercises [3].

### Existing Evidence Base

The integration of computerized interventions into mainstream mental health services offers a number of benefits, including the means to increase capacity in mental health services, reduce costs, and improve convenience to therapist and client [4,5]. In addition, online interventions have the ability to surmount geographical barriers as well as barriers associated with stigma that may prevent clinic attendance [6,7]. Not only is it important to establish that computerized interventions do what they are intended for, but it is also crucial that they are designed to be engaging, usable, and acceptable to those who need them [8], otherwise these factors may mask the effectiveness of the intervention. Much of the literature is concerned with 2 themes of enquiry which are central to the development of a clinically useful body of knowledge related to computerized psychotherapeutic interventions.

#### Clinical Efficacy

The first theme relates to the actual clinical efficacy of the computerized intervention. This line of enquiry seeks to ascertain if completion of the intervention leads to any significant increase in well-being or decrease in distress for the user. Typically, this manifests as an outcome study that assesses symptomatology and other clinically relevant variables preintervention and postintervention. Results are promising; in a comprehensive meta-analysis of the effectiveness of Internet-based psychotherapeutic interventions, Barak et al [2] reported a similar effect size to that expected with more traditional face-to-face therapy. A review of the literature concerned solely with depression tallies with this [9]. Multiple studies and meta-analyses conclude that computerized and online psychotherapeutic interventions (most CBT-based) offer a promising and potentially effective treatment of depression and anxiety [6,7,10-12]. Many researchers highlight, however, that caution is advised in generalizing these conclusions too widely or too prematurely, and emphasize that more systematic research is needed to clearly delineate the precise factors that contribute to the effectiveness of online and computerized interventions. For instance, efficacy can vary according to factors, such as level of therapist support, the presenting difficulty of the client, the length of treatment, the age of the client, the media and technology used to deliver the program, and the interactivity of the program [2,6]. Even seemingly small differences among programs and their delivery can have implications for outcomes. In particular, research suggests that even a minimal amount of therapist support can increase the efficacy of an online or computerized program [7,9], particularly in the treatment of depression [5]. Some research suggests both supported and unsupported online interventions may be effective [13,14]. A recent meta-analysis of online interventions for depression highlighted the superiority of supported interventions in terms of outcomes, but noted that unsupported interventions may also have a useful role to play in treatment provision [15]. At present, it seems reasonable to conclude that some form of support has an added beneficial effect for program outcomes, but the absolute necessity of therapist support, the optimum level of such support, or its precise nature remains to be established [5,13,15]. It is unclear which elements of the guidance or support are responsible for any observed benefits, with Andersson et al [16] suggesting that positive reinforcement for work completed by the client may be the most important contribution of the person supporting the online intervention.

#### Engagement

The second theme of enquiry relates to the issue of how engaging computerized interventions are and how willing clients are to use them as they are intended. Related to this, researchers have also begun to examine the characteristics of the individuals most likely to both engage with and benefit from a computerized intervention [1,17,18]. Although attrition from psychotherapy is an issue that affects services offered in more traditional ways [19,20], it has been repeatedly highlighted as a significant issue in online interventions in both research and practice [14,21-23]. There are a number of factors that may predict engagement with and adherence to online interventions, including factors related to the program itself and how it is delivered [2,23,24], as well as characteristics of the program users, such as age, gender, education, duration and severity of psychological distress, and personal psychological factors [20,23-26]. To improve engagement, a number of researchers have successfully included direct therapist support alongside the Internet intervention, such as motivational interviewing and regular telephone conversations with therapists [7,9], suggesting that human contact is vital for achieving high levels of engagement. A meta-analysis of the literature has suggested that although overall dropout rates from online programs for depression may exceed 50% [15], this can be substantially reduced by the addition of some form of support or guidance [14].

### A Therapist-Supported Online Program

We can see from the literature that the efficacy of online programs can vary as a function of delivery and context, and that difficulties remain surrounding engagement and attrition. This motivates development and exploration of innovative strategies for improving engagement in realistic service environments. To this end, we present an initial clinical evaluation of a highly interactive therapist-supported Internet-based intervention for depression, namely the Mind Balance program. We report the engagement patterns of clients using the therapist-supported program, and outcome data on changes in self-reported depression symptomatology following use of the program.

### Program Content

Mind Balance is a 7-module online CBT-based intervention for depression, delivered on a Web 2.0 platform using media-rich interactive content. The structure and content of the program modules follow evidence-based principles of a traditional CBT program, incorporating ideas from mindfulness. The content of each module is described briefly in Table 1. Each module is structured in an identical way and incorporates introductory quizzes, videos, informational content, interactive activities, as well as homework suggestions and summaries. In addition, personal stories and accounts from other clients are incorporated into the presentation of the material.

### Engagement Strategies Employed Within the Program

The Mind Balance program employs a number of innovative engagement strategies for improving the user experience, described subsequently. These features are also presented in the SilverCloud Platform Overview video (Multimedia Appendix 1). The design emerged from an extensive user-centered design process and was refined through a number of formative evaluations.

#### Personal

**Figure 1:**
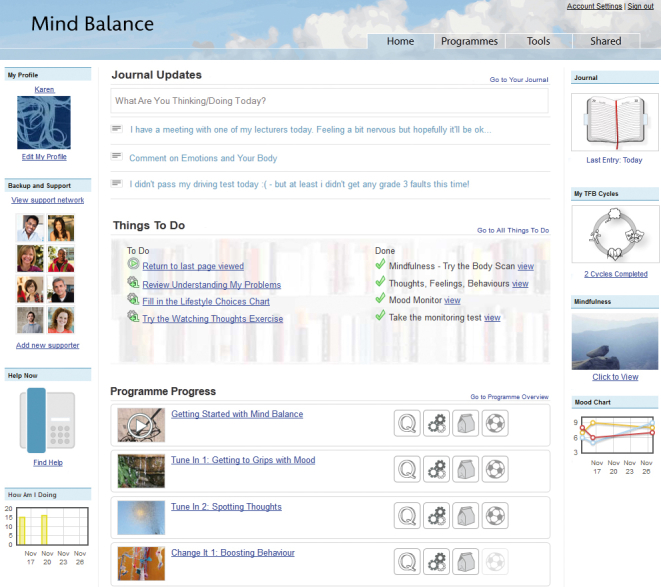
Program home page.

The client has their own secure home page (Figure 1), which is about who they are and where they are in the program. For example, the client can fill in a profile with basic information about themselves, such as age and interests. This establishes a sense of ownership, and provides useful information for the therapist, allowing them to provide feedback that is more personal. The home page is intended to provide a reflective space; the client can document their thoughts and feelings, and these can be elaborated on within the journal application, which also acts as the vehicle for therapeutic writing exercises. The user has actions suggested to them, and as they complete modules of the program, their achievements are noted. Users are free to access the modules in any order they wish, in either a linear or nonlinear manner, contributing to a sense of empowerment. A range of satellite applications are provided along with the central content, such as a goal-setting application, which can be used independently of the program content. Applications are released as the user goes through the program, with the intention of maintaining engagement by introducing new features over time and not overwhelming the client initially. Clients can also control which applications appear on their home page.

#### Interactive

**Figure 2:**
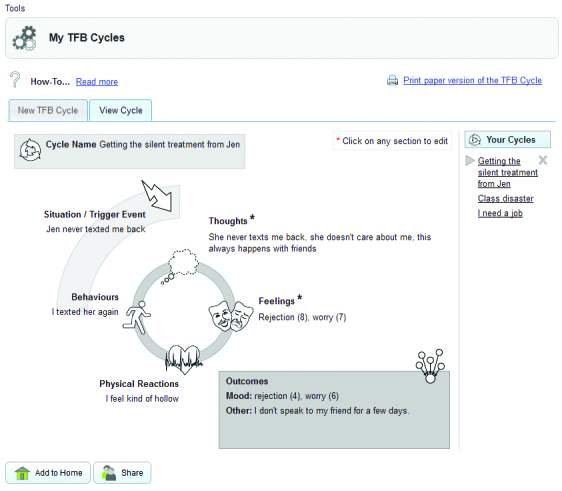
Example of an interactive exercise: thoughts, feelings, and behaviors.

The program includes a number of interactive elements and graphical exercises (an example is shown in Figure 2), which are aimed at engaging clients with the therapeutic content, for example, reflecting on their own thinking. Clients also have the ability to respond to content, indicating whether they like it, and also to comment on it. The client is provided with immediate feedback wherever possible; for example, when a charting exercise, such as a mood chart, is completed, the application item is graphically updated on the home page. Likewise, items are ticked off on the to-do list when completed, and achievements unlocked in each module summary.

**Table 1:** Mind Balance: description of module contents.

Module name	Brief description
Getting Started	Outlines the basic premise of CBT, some information about depression, and introduces some of the key ideas of Mind Balance. Users are encouraged to begin to chart their own current difficulties with depression.
Tune In 1: Getting to Grips with Mood	Focuses on mood monitoring and emotional literacy. Users can explore different aspects of emotions, physical reactions, action and inaction, and how they are related.
Tune in 2: Spotting Thoughts	This module focuses on noting and tracking thoughts. Users can explore the connection between their cognitions and their mood, and record them graphically.
Change It 1: Boosting Behavior	This module focuses on behavioral change as a way to improve mood. Ideas about behavioral activation are included, and users can plan and record activities, and chart their relationship with their mood.
Change It 2: Challenge Your Thoughts	This module supports users to challenge distorted or overly negative thinking patterns, with thought records, as well as helpful coping thoughts.
Change It 3: Core Beliefs	This module outlines the role that deeply held core beliefs can play in mood and depression. Users can use a range of interactive activities to identify, challenge, and balance any unhelpful core beliefs.
Bringing It All Together	Users are encouraged to bring together all the skills and ideas they have gathered, note their personal warning signs, and make a plan for staying well.

#### Supportive

Each client has an assigned therapist, who provides weekly reviews of their progress on the program. This support is asynchronous, whereby the therapist sets a date to review their client’s progress, and they do not provide feedback, support, or contact outside this time. This is an important program feature in terms of maintaining the therapeutic boundary in the online space. The therapist can support multiple clients, logging in once weekly for instance, and reviewing the work of all their online clients within an allocated time period. Such asynchronous online contact may be logistically easier to implement for many services compared with motivational interviewing and telephone support, and should be more attractive to users who are unwilling to have direct contact with a service. The system supports the exchange of messages between the client and therapist, but goes beyond email because the client is encouraged to share their content (such as completed exercises and comments) with their therapist (see Figure 2). This shared content allows the therapist to respond in a more personal way and provide guidance as well as encouragement to keep using the program. Adherence information is also available to the supporter, and they can keep track of the client’s progress. This is all personally sensitive information; for this reason, a shared view is provided in the client interface where they can see the therapist’s view of their data. By making the visibility of their data to the therapist more transparent, as well as the ability to explicitly change the sharing status of data, the client is provided with a greater sense of control while facilitating a meaningful interaction with the therapist.

#### Social

Although group therapy and peer group support are well established, introducing contact with other clients within any online system raises a number of ethical concerns regarding the possibility for unhelpful or negative content or communications. As a first step, the client can see anonymous indications of other people in the system. The intention is to reassure clients that they are not alone in experiencing difficulties and that many other people have experienced similar problems and overcome them. Users can respond to content by indicating that they “like” it, and can see how many other people liked it, helping to reduce the sense of isolation. More detailed shared content (such as tips and ideas) is subject to therapist moderation.

A key innovative aspect of the program is the way in which these different strategies are combined within the overall design. For example, the interactive features of the program generate content which can be shared with the therapist (enhancing the quality of feedback which can be provided), and in some cases with peers (further enhancing the experience of other users).

## Methods

A service-based design was used to assess engagement with the Mind Balance program delivered by the mental health service of an Irish university (Trinity College, Dublin). As a first exploratory trial, it was felt that a service-based design would be informative regarding the potential of the program to engage clients, provide initial evidence regarding the potential effectiveness of the treatment, and establish the feasibility of incorporating low-intensity online therapist support in a real-world environment. Part of the appeal of the program to this population was expected to be the relative anonymity of the online mode of delivery; therefore, face-to-face contact was not required to sign up for the program. The protocol for use was kept as close as possible to the actual practice for use of online interventions within the service; this degree of fidelity was borne out by the subsequent incorporation of the program into the day-to-day practice of the service following the same protocol. Client log-ins and use of program features were automatically monitored. Changes in symptoms of depression were also assessed through measures taken before and after use of the program. Ethical approval was secured from the Research Ethics Committee of Trinity College prior to the study.

### Recruitment

Participants were recruited in 2 ways as the program was rolled out, in parallel with the usual practice of the counseling service.

#### Self-Sign-Up

Recruitment began in January 2011, with all first- and third-year students emailed through the university emailing system to inform them of the opportunity to receive this online intervention as part of a research study. In September 2011, all fourth-year and postgraduate students were similarly emailed. Finally, in January 2012, all first years were again emailed to inform them of this opportunity. This rolling recruitment model was used to manage the numbers of students simultaneously availing of the intervention because of limited therapist resources. Specific-year groups were chosen at various times based on existing records of service engagement patterns in the student counseling service. Participants were informed that they would be required to register with the student counseling service in order to avail of the program. Interested students were instructed to follow a Web link embedded in the text of the email and were directed from there to the sign-up page. Students could also access this Web link from the University’s online mental health portal. Once an individual had navigated to the sign-up page, they were presented with a comprehensive information sheet and an electronic consent form. Following confirmation of informed consent, students were required to complete the preprogram measures electronically and were then free to explore the online program as they wished. Postintervention measures were sent out automatically after completion of the final review.

#### Directly Added by Face-to-Face Therapist

A number of participants were referred directly to the program by staff in the counseling service. Those receiving face-to-face therapy in conjunction with the program were excluded from the research sample.

### Participants

Participants were 80 students who identified themselves as having an existing difficulty with low mood, mean age 23.29 years (SD 4.84) and 69% female, who were all part of the population supported by the student counseling service. All of the participants were registered students. Demographic details for the main and subsample are detailed in Table 2.

**Figure 3:**
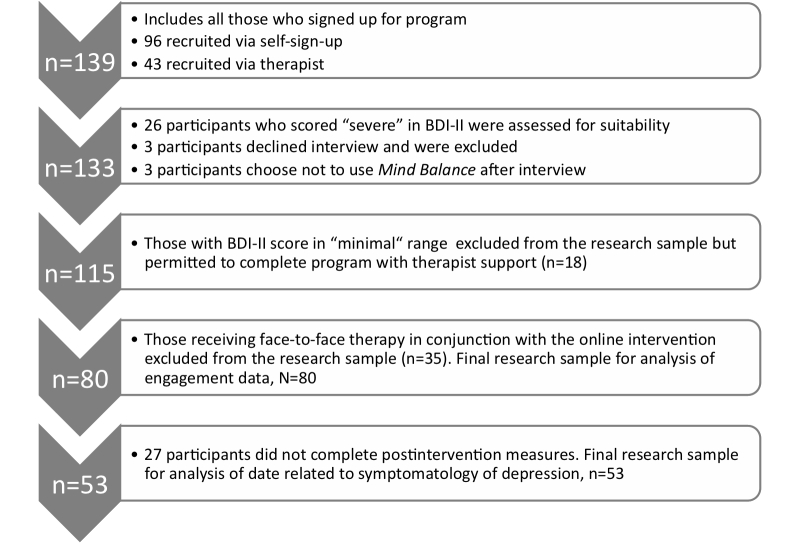
Exclusion pathway to determine sample and sub-sample.

Inclusion criteria were as follows: (1) at least 18 years of age, (2) having at least self-reported mild symptoms of depression with a Beck Depression Inventory-II (BDI-II) score ≥14, and (3) have received no face-to-face therapy since program commencement. A breakdown of how this final sample was reached through exclusions from the initial sample is provided in Figure 3.

**Table 2:** Demographic details of the sample and subsample for pre-post analysis.

Group	Age, mean (SD)	Age range	Male, n (%)	Female, n (%)
Overall sample (N=80)	23.28 (4.84)	18-46	25 (31%)	55 (69%)
Pre-post subsample (n=53)	22.69 (5.17)	18-46	13 (25%)	40 (75%)

#### Ethical Considerations

Telephone and email contact details for support services (including the counseling service, public emergency services, and relevant non-government organizations) for crises or out-of-hours emergencies were provided to users in the “help now” section of the website, which was visible at all times regardless of the page the user had navigated to. Any individual who scored 29 or more on the BDI-II or who scored 2 or more on BDI-II item 9 (suicidality) in their online screening was automatically alerted that a counselor would get in touch with them. They were telephoned within 1 working day to invite them for an interview, and contacted by email if they could not be reached by telephone. The screening interviews were carried out by a counseling psychologist and focused on the client’s own description of the problem, including duration of the problem and their understanding of the causes, their psychiatric or mental health history, current alcohol and drug use, current health and functioning (sleeping, eating, attending lectures, keeping up with commitments, etc), and current support network. A clinical decision was made on the basis of this assessment in relation to whether the client could go ahead and use the online program on a standalone basis. Alternatively, it was recommended that they attend face-to-face counseling instead of or in conjunction with the online program. Three clients declined to attend for interview and were excluded. Three others chose not to use the program following the interview and were excluded. Those receiving face-to-face therapy combined with the program were not included in the research sample presented here. Although the primary concern was client safety, the approach taken was one which would be applied in the normal practice of the service.

### Measures

#### Engagement

The online system collected anonymized descriptive information relating to engagement and usage. Data collected included the number of sessions completed, mean time spent on the program, average number of sessions per client, and average length of a session. A session was defined as an instance when a client logged on to the system. Session time estimation will always be an imperfect calculation because users may be interrupted or take breaks within a session, and may not formally log out of the system. All client activity within the system, such as reading a content page, saving a journal entry, or updating an activity, was logged with a time stamp. Starting with the log entry of the client logging on, the total time was calculated by adding up the time that elapsed between each subsequent log record. On its own, this yields a result vulnerable to overestimation of session time. To avoid counting periods when the user was not actively engaged with the system, any interactions taking longer than 30 minutes were counted as 1 minute. Any period of inactivity longer than 3 hours started the count on a new session, rather than extending the time of the current session. Use of different program components was also measured. Data related to therapist reviews were also collected. In the interests of examining engagement and usage patterns in a fair and meaningful way, all 80 participants were included in that analysis regardless of whether or not they had completed the BDI-II postintervention. Dropout is analyzed in terms of the last time a participant used the system.

#### Symptomatology of Depression

The BDI-II was used to assess the severity of self-reported symptomatology of depression preintervention and postintervention. Cut-off scores suggested for the BDI-II are minimal symptoms: 0-13; mild symptoms: 14-19; moderate symptoms: 20-28; and severe symptoms: ≥29 [27]. Reliability, validity, and test-retest reliability have been established across various populations and cultural groups [27-31].

All data were collected and stored electronically on a secure server.

### Program Delivery

Following initial sign-up, clients were assigned a therapist who would support them online. This happened automatically and randomly in the case of those clients recruited via self sign-up. In the case of those who signed up following a recommendation by a face-to-face therapist, the same therapist was assigned as their online supporter. While the program suggested that clients complete 1 module per week, clients were free to dictate the pace and order of their work. Therapists were asked to provide 7 weekly reviews of each client’s work. Allowing for 1 final session for the client to consider the final review, this corresponds to a target of 8 sessions per client, and also matches the target of 8 sessions in the program previously used within the service evaluated in Richards [32]. The service scheduled 1 hour per week for the 6 participating therapists to complete their Mind Balance reviews. The program content was identical for each client, but each therapist was free to provide reviews congruent with their own therapeutic persuasion. Therapists were given training and a treatment manual containing a broad guide of how to respond in their reviews (supporting progress, giving encouragement, specific feedback on activities shared), but could be flexible if they wanted to add to this. For the most part, this was only expected to happen when the users shared large amounts of personal content. In these cases, therapists were advised that this was not email counseling, and so very lengthy detailed responses were not advised. A dashboard interface was provided to therapists that gave an overview of their clients and the overall level of engagement of each, with additional detailed engagement information and shared data available for each client. In the interests of transparency, this information was available to clients under a “shared” tab in the interface. When the client was not engaging with the system, more generic messages could be used, and a template system was provided to facilitate this. All therapists held postgraduate qualifications and consisted of 3 counseling psychologists; 1 CBT therapist; 1 marriage, family, and child counselor; and 1 psychotherapist. Clients were informed at each review of the precise date for the next review. If the therapist wished, they were free to continue to provide reviews for their client past the seventh recommended review, if they saw this as clinically justified, again with the aim of allowing a natural service-based pattern of use. Email technical support was available to both clients and therapists.

## Results

### Engagement

**Figure 4:**
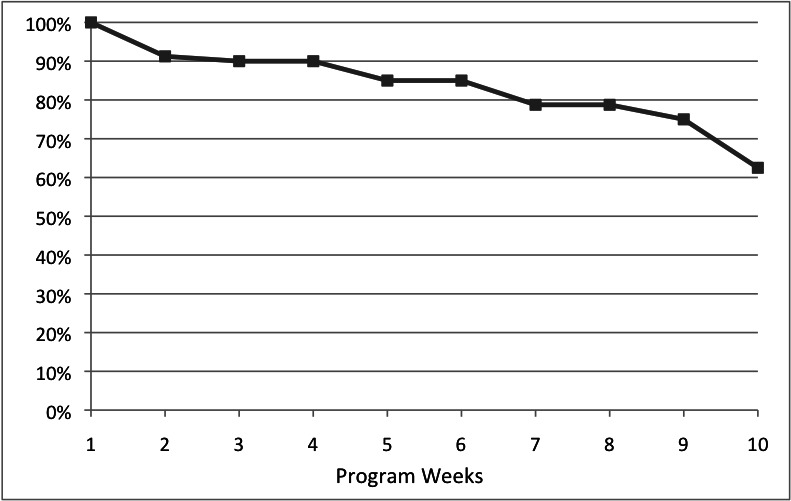
Dropout of clients over time.

Figure 4 illustrates the level of engagement with the program for the overall sample (N=80). This graph depicts the number of users who were engaged at a given week or later, and conversely the week at which users dropped out. Looking at the graph, it is evident that relatively few users dropped out in the initial weeks of the program, and 79% were engaged at the target week 8 or later.

Table 3 presents key descriptive statistics relating to program usage. An examination of the log data reveals that 50% of reviews were read by the recipient (client) within 24 hours. Therapists reported that reviewing a client’s work took 10 to 15 minutes per review on average, varying by the degree of engagement and content shared. The template feature allowed therapists to quickly send more generic encouragement to clients not logging in to the program.

Viewing the engagement data by BDI-II category (Table 4), clients in the severe category spent more time on average in the program than clients in the mild and moderate categories. A demographic breakdown of those reaching the target of 8 sessions is presented in Table 5.

**Figure 5:**
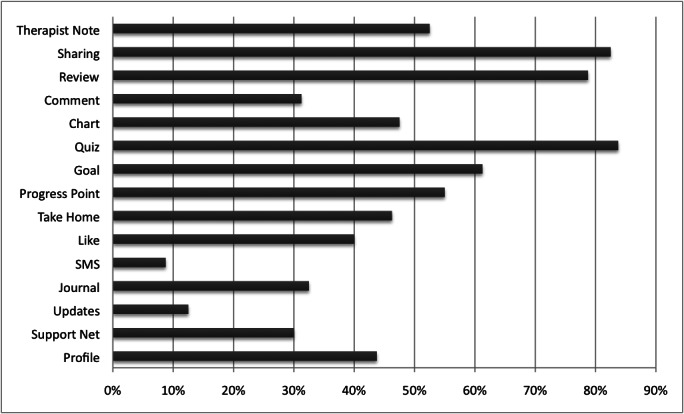
Client usage of program features.

Usage of different program features is illustrated in Figure 5. These refer to the percentage of clients sending notes to the therapist, sharing content with the therapist, viewing the therapist review, commenting on the content, performing a charting exercise, completing a psychoeducational quiz, setting a goal within the goal-setting tool, giving feedback on what they thought of a module, entering their own take-home point, “liking” content, sending brief updates via short message service (SMS) text messaging, writing within the journal, making brief updates from the home page, filling in the backup and support network, and filling in personal information in the profile.

**Table 3:** Usage statistics for overall sample (N=80).

Statistic description		Mean (SD)	Range
**Time (minutes:seconds)**			
	Length of time per session	11:35 (18:00)	0:00-125:11
	Total time spent on program	151:09 (141:48)	0:00-712:09
**Sessions**			
	Number of sessions per user	11.95 (9.79)	1-53
**Reviews**			
	Number of reviews per client	7.4 (4.2)	2-33

**Table 4:** Usage by Beck Depression Inventory-II (BDI-II) category for the overall sample (N=80).

BDI-II category	Mild (n=19)	Moderate (n=41)	Severe (n=20)
Time (minutes:seconds), mean	120:33	132:43	231:37
Sessions, n	9	10.6	18
Target of 8 sessions, %	47	54	95

**Table 5:** Demographics of session target completers and noncompleters.

Number of sessions	Age (years), mean (SD)	Age range	Male, n (%)	Female, n (%)
≥8 sessions	22.6 (5.1)	18-46	13 (26%)	37 (74%)
<8 sessions	24.5 (4.4)	18-34	12 (40%)	18 (60%)

### Change in Symptomatology of Depression

The reliability of the BDI-II was assessed using Cronbach alpha and was found to be in the acceptable range for both the overall sample (N=80) and the subsample (n=53), being Cronbach alpha=.78 and Cronbach alpha=.79, respectively. This indicates satisfactory internal reliability for the BDI-II for the present sample. Table 6 presents the mean BDI-II scores for the present sample both preintervention and postintervention, as well as results of the repeated measures *t* tests of difference.

As illustrated in Table 6, results indicate a statistically significant (*P*<.001) decrease in symptomatology of depression, as assessed by the BDI-II, from preintervention to postintervention. Cohen’s *d* was calculated as *d*=1.17, suggesting a large pre-post effect size for BDI-II [33]. An independent samples *t* test revealed no significant difference between preintervention BDI-II scores of those who completed postintervention measures (n=53) and those who did not (n=27), *t*_78_=–0.382, *P*=.70. Table 7 outlines the number and percentage of participants in each category of severity of symptomatology preintervention and postintervention for the subsample (n=53) only. Postintervention, 74% of participants’ (n=39) BDI-II scores had reduced and placed them in a lower category of severity (eg, from moderate to mild) than that suggested by their preintervention BDI-II score.

**Table 6:** Comparison of BDI-II scores preintervention (pre) and postintervention (post) and repeated measures *t* test.

BDI-II score	Pre, mean (SD)	Post, mean (SD)	*t*_52_	*P* value
Overall sample (n=80)	25.71 (7.80)	—	—	—
Subsample (n=53)	25.47 (7.95)	15.53 (9.06)	7.70	<.001
No post-BDI (n=27)	26.19 (7.64)	—	—	—

**Table 7:** Participants in each severity category preintervention and postintervention (subsample only).

BDI-II category (score range)	Preintervention, n (%)	Postintervention, n (%)
Minimal (0-13)	0 (0)	28 (53)
Mild (14-19)	13 (25)	7 (13)
Moderate (20-28)	23 (43)	11 (21)
Severe (29+)	17 (32)	7 (13)

## Discussion

### Engagement

Poor treatment adherence and dropout have been repeatedly highlighted in the literature as significant issues to overcome in the delivery of Internet interventions [20,21]. Looking at the present study, it is evident that dropout for this interactive therapist-supported program is gradual up to the end of the supported treatment period. Viewing dropout in terms of the number of participants not engaged at session 8, the dropout rate for the present study stands at 37.5%. This compares favorably with the reported dropout rate of 74% for an unsupported program [32]. Given that the interventions were delivered in the same setting and with a similar population, this implies that delivering online programs incorporating online therapist support may enhance engagement and completion rates. This concurs with previous research [23,24] that suggests characteristics of program delivery, such as the presence of therapist support, may significantly influence client engagement with online programs. As a further point of reference, internal statistics within the service put average session attendance for face-to-face treatment at 3.49 and 3.73 sessions over the 2 years that this study took place. Approximately 12% of clients attended for 8 sessions or more.

On the demographic breakdown, we must note that this is a relatively homogeneous population compared with what may be seen in other environments. The student population supported by the service has a majority of women (59% in 2010/2011), and the greater number of women signing up for the program mirrors the use of the service generally. The incidence of depression also differs by gender. The size of the sample for male participants precludes us from making any statements regarding the lower number of males completing the target, but this issue would be worth investigating in future studies.

We can see from Figure 5 that key features regarding therapist support, such as sharing, therapist notes, and therapist reviews, were used by most users. However, social features, such as the like button, also received significant numbers of users, and the progress point, take-home point, and goal-setting features, which required more active participation, were also used by many users. The SMS text messaging feature that allowed users to make brief updates via mobile phone was only used by 7 of 80 users (9%). The Mind Balance program did not place emphasis on these updates, and further work is needed on how best to integrate mobile support into this type of intervention. Another interesting direction for analysis would be to investigate the relationship between viewing the content of particular modules and usage of the other features of the program. Overall, we can see from this data that the interactive features of the program were used to a significant extent, and that the system was not used simply as a multimedia delivery medium. Features within the program intended to improve engagement cover many, but not all, of the persuasive framework elements presented in Kelders et al [34]. As such, there are further design opportunities to be explored within this form of intervention, particularly in the area of social support.

### Therapist Support

The primary aim of online interventions is to maintain clinical gains while reducing the amount of therapist time required per client. It is worth reflecting on the precise nature of the support needed to maintain an optimum pre-post clinical effect as well as satisfactory completion rates [5]. A number of studies have used therapist support that is reasonably time consuming (eg, motivational interviewing or weekly telephone calls), which may counteract some of the benefits of using online interventions. Even for low-intensity interventions using telephone support in which the overall amount of therapist contact time is similar (eg, 64 minutes [35]), it can be argued that the overhead in the online case is likely to be lower because of the lack of scheduling problems, and the flexibility available to the therapist in terms of when to perform the reviews. The system integrated therapist support into the same online space to maximize efficiency and ease-of-use for the therapist, while still maintaining the clinical benefits of therapist input. The fact that pre-post outcome effect size and completion rates for the present study were satisfactory despite using a relatively low level of therapist input is encouraging. Investigating the cost-effectiveness of this form of support would be an interesting direction for future work.

The nature of the support given by the therapist also deserves further exploration. Taking persuasive technology as encompassing both direct interaction with a computer system and computer-mediated communication [34], there is a need to explore and validate models for Internet interventions [36] that are useful for design and that encompass this human support. An initial theoretical model for human-supported eHealth interventions has been proposed by Mohr et al [37] that provides a number of testable hypotheses. This model also generates a number of useful suggestions regarding the nature of the support or coaching provided to clients. Exploring and refining such a model together with emerging best practice surrounding this program constitutes a promising direction for future work. However isolating the effects of a large number of interdependent program features is a significant challenge and motivates the development of new approaches that leverage finer grained analysis of usage data. Qualitative exploration of the issues surrounding the perception of a therapeutic relationship with both the system and the supporter, and the most appropriate forms of feedback would also be valuable. Previous work suggests the impact is spread across several factors that both reduce and enhance engagement [38].

### Outcomes

A main focus of the development of the program was to improve client engagement; therefore, the outcome data are encouraging, with a significant reduction in depressive symptomatology following program completion, with a pre-post effect size estimate of *d*=1.17. This concurs with the current body of literature, which does suggest a reasonably robust pre-post outcome effect size for online CBT-based interventions [7]. As Andersson and Cuijpers [9] point out, it is important to distinguish between interventions that are therapist supported and those that are not when estimating efficacy. Pre-post clinical outcome effect sizes for therapist-supported programs have been estimated at *d*=0.61 and *d*=1.0 in meta-analyses by Andersson and Cuijpers [9] and Spek et al [7], respectively. Thus, the pre-post effect sizes for the present study are consistent with those found with existing comparable interventions.

### Recruitment

Recruitment for the study also illustrates the appeal that online interventions may have for some clients. All students are eligible for free face-to-face treatment, but the offer of online treatment led many people with clinically significant symptoms of depression to make contact with the service for the first time. This suggests that therapist-supported online interventions, such as Mind Balance, may attract people who are reluctant to engage with face-to-face treatment; hence, improving access to psychological interventions for those in need of them.

### Limitations

The findings of the study are promising, but they should be interpreted with caution. Although the results were statistically significant, the modest sample size motivates continued long-term data gathering. Further replication of the research is also needed. Similarly, the absence of a control group does not allow us to draw specific conclusions with regards the efficacy of the program in relation to spontaneous symptom improvement [39]. As a repeated measures experiment, care should also be taken when comparing to independent group effect sizes [40]. A further controlled study should also examine the maintenance of gains at follow-up, as this was a further limitation of this initial study. Given the service-based nature of the study, clients included those who had contact with the service before commencing the program, which is an additional potential confound. However, in terms of representing a realistic client mix, this is a group that needs to be supported by the service, and for whom online treatment options should be available.

The study did not automatically exclude potential participants who reported symptoms of depression in the severe range. Although this results in a sample that may differ from that in several comparable studies, it mirrors a service-based pattern of use. The level of engagement with the program exhibited by this group was high, and together with the positive outcome data, suggests that clients along the full span of the symptom severity spectrum may benefit from supported online programs. This service-based study provides an example of how online interventions may be used and fit in with existing mental health services in this setting. The program is now embedded within the day-to-day practice of the service, following the same procedure described here, illustrating the ecological validity of the deployment within the study. Knowledge gained through the study has also been fed into the development of a more detailed treatment manual for supporters to be used in future trials. All data related to time spent on the program, modules viewed, exercises completed, and so on were collected electronically. This ensured a high level of accuracy in determining the level of system usage because there was no reliance on self-reports of time spent on the program.

### Conclusion

In this paper, we have presented a study of a new and innovative program for online CBT integrating a range of features intended to increase client engagement. Both client and therapist users were able to use the system successfully, and they made use of a range of program features. The findings suggest that a low level of asynchronous online therapist support is a promising avenue for the development of online interventions, when appropriately integrated into the delivery of the online intervention. As a new mode for delivery of online guided self-help, it contributes to the exploration of the optimum level and nature of therapist input needed to achieve clinical gains in an accessible manner and provides an indication of the many possibilities within the design space for such systems. It strongly motivates exploration of issues of clinical efficacy within a service-based randomized controlled trial and of the potential effectiveness of this form of interactive delivery for other types of intervention.

## Multimedia Appendix 1

Demonstration of innovative program features.

## Abbreviations

BDI-II: Beck Depression Inventory-II
CBT: cognitive behavioral therapy
SMS: short message service
